# A 44 year-old lady with chronic renal disease and intractable ulcers: a case report

**DOI:** 10.1186/1755-7682-2-22

**Published:** 2009-07-31

**Authors:** Thejeswi Pujar, Irene M Spinello

**Affiliations:** 1 Kern Medical Center, 1830 Flower Street, Bakersfield, California 93305, USA; 2 David Geffen School of Medicine at UCLA, Los Angeles, California, USA; 3 Critical Care and Pulmonary Services, Kern Medical Center, 1830 Flower Street, Bakersfield, California 93305, USA

## Abstract

Calciphylaxis is a rare but potentially fatal condition occurring in patients with end stage renal disease on dialysis. Due to interplay of various factors, disturbances occur in the metabolism of calcium and phosphate leading to calcification within the vessel walls. The net result is tissue ischemia and necrosis. Clinically this presents as painful non-healing skin ulcers, which contribute to significant morbidity and mortality due to septic progression of the lesion. In this case report, we highlight the rapidly progressive nature of this disease, its etiopathogenesis and the role of early diagnosis in preventing life-threatening complications.

## Introduction

A 44-year-old Caucasian female with chronic renal failure receiving dialysis for the past one year presented to our hospital complaining of two weeks of diarrhea and worsening nausea. She was scheduled to receive dialysis treatments thrice weekly but had continuously missed her scheduled appointments. At the time of her initial presentation she had not been dialyzed for the past two weeks. Her past medical history was also significant for type 2 diabetes mellitus, hypertension, dyslipidemia, anemia of chronic disease and bronchial asthma. She had a history of cholecystectomy and cesarean section. There was a strong family history of hypertension, coronary artery disease and diabetes mellitus. She denied smoking, alcohol intake or use of recreational drugs. Her current medications included Vicodin, Actos, insulin and albuterol HFA. She had history of allergy to Penicillin.

### Physical examination

On examination she was disabled with morbid obesity and in no apparent distress. There was no evidence of pallor, cyanosis or jaundice. Her vital signs revealed heart rate of 106/min, BP 140/78 mm of Hg, temperature 95.4°F, respiratory rate 18/min, and saturating at 99% on room air. She had a surgically implanted left subclavian dialysis catheter. HEENT exam revealed mild dehydration. Systemic examination was significant for mild epigastric tenderness without guarding or rigidity. There were areas of ecchymoses over inner thighs. Peripheral pulses were normal.

### Laboratory tests

Laboratory investigations revealed potassium of 3.1 mg/dL, bicarbonate 22 mEq/L, BUN 74 mg/dl, creatinine 6.3 mg/dl, anion gap 20 mEq/L, phosphorous 7.2 mg/dL, serum calcium 9.1 mg/dL, magnesium 1.5 mg/dL, hemoglobin 10.5 g/dL, hematocrit 30%, serum iron 29 mcg/dL, TIBC 84 mcg/dL, normal amylase, lipase, thyroid function tests and liver function tests except albumin of 1.4 g/dL. EKG revealed sinus rhythm with RBBB. Chest radiograph was normal.

### Hospital course

**Figure 1:**
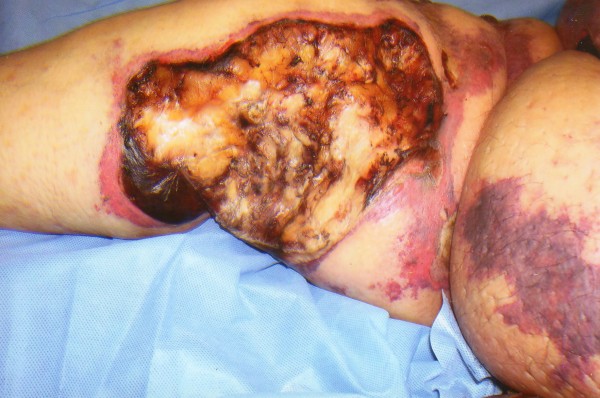
A large inner thigh ulcerated lesion with peripheral necrotic edges surrounded by areas of violaceous skin discoloration.

**Figure 2:**
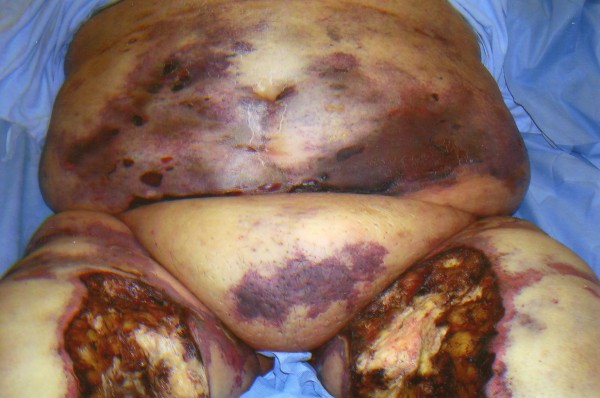
**Extensive involvement of the thighs and lower abdomen**.

The patient was admitted for hemodialysis, correction of electrolyte imbalance and for evaluation of diarrhea. Patient was started on epoetin alfa for anemia, sevelamer hydrochloride (Renagel) for hyperphosphatemia and heparin for thromboprophylaxis. Over the next several days, the diarrhea resolved but patient developed worsening body aches and thigh pain. Blood test revealed leukocytosis at 19.7 × 10^3^/mm^3 ^with neutrophilia at 13.7 × 10^3^/microL, hemoglobin of 7.9 g/dL, hematocrit 25.3%, without any evidence of bleeding. Stool tests were non-contributory. Blood cultures revealed methicillin sensitive staphylococcus aureus (MSSA) from the peripheral intravenous line as well as a surgically implanted long-term dialysis catheter (Ash-cath). The site of Ash-cath was erythematous; therefore, it was removed. The patient was started on ceftriaxone. Over the next couple of days the patient's ecchymoses over the thighs began to worsen accompanied with induration, bullae and necrosis. The lesions started spreading to the lower abdomen as well (Figures 1 and 2).

Given the typical skin lesions in a patient with DM and end-stage renal disease (ESRD) on hemodialysis, with an increased calcium phosphate product (Ca × PO_4_) at 65.5 mg^2^/dL^2^, a diagnosis of Calcific Uremic Arteriopathy (CUA) was made. Her other risk factors included morbid obesity, history of treatment with calcium based phosphate binder Phoslo, hypoalbuminemia and hyperphosphatemia. Further coagulation studies and ANA were normal, therefore, ruling out a possibility of hypercoagulable conditions or vasculitis. Plastic surgery service performed multiple debridements of the lesions over the thighs. Biopsy of the lesions revealed chronic skin gangrene. Tissue cultures grew vancomycin resistant enterococcus (VRE) and MSSA. Appropriate antibiotic therapy was started based on these sensitivities and daily dialysis treatments continued. But despite these measures, her skin lesions continued to worsen. She eventually developed severe sepsis and septic shock with disseminated intravascular coagulation (DIC) and passed away.

## Discussion

Calciphylaxis, also called Calcific Uremic Arteriopathy (CUA), is a rare but life-threatening condition characterized by vascular calcification and cutaneous necrosis usually seen in patients with ESRD on long-term dialysis. In this population, the prevalence of CUA is 4.1% [1]. It was first described in 1962 by Selye in nephrectomized rats [2]. Since 1976 this condition has been reported primarily in patients with ESRD [3]. Although its pathogenesis is unclear, hypercalcemia, hyperphosphatemia, with an elevated calcium phosphate product, and secondary hyperparathyroidism have been implicated in its pathogenesis. The Ca × PO_4 _product is derived by multiplying total calcium by phosphate and should normally be less than 30 mg^2^/dL^2 ^(22.2 – 45.5 mg^2^/dL^2^).

There are many risk factors associated with this condition as highlighted in Table 1[4-11]. Of these factors, some – such as Vitamin D and PTH – are called sensitizers and others – such as trauma and iron dextran injections – are called challengers. It is the interaction of sensitizers and challengers that lead to abnormal calcium and phosphate metabolism with calcification of small and medium sized arteries and arterioles leading to occlusion of the vessel and tissue infarction.

**Table 1:** Risk factors for Calciphylaxis.

**Patient-related factors**	Female sex; Caucasian race; Morbid obesity (BMI > 30 kg/m^2^); Protein malnutrition (serum albumin ~2 gm/dL); Recent weight loss (>10% in 6 months).
**Biochemical factors**	Hypercalcemia >10.5 mg/dL; Hyperphosphatemia (>6.5 mg/dL); Calcium × Phosphate product (Ca × PO_4_) > 55 mg^2^/dL^2^; Alkaline Phosphatase 10 IU above normal; Hyperparathyroidism (primary or secondary)

**Disease factors**	ESRD on replacement therapy; Diabetes Mellitus; Cirrhosis of liver; Hypercoagulable states (e.g Protein C and S deficiency); Malignancy, Multiple myeloma, Lymphoma, Cholangiocarcinoma.

**Medication-related factors**	Warfarin therapy; Use of steroids; Use of vitamin D & calcium based phosphate binders; Drug infusions such as parenteral iron therapy; Trauma (e.g. subcutaneous insulin injections).

### Clinical features

Patients with calciphylaxis present with acute onset of exquisitely tender cutaneous nodules or plaques with violaceous or black discoloration. The distribution of these areas can be proximal or distal. In case of proximal distribution, the cutaneous findings appear in areas with high content of adipose tissue such as trunk, thighs and buttocks. This variant carries poor prognosis, while the distally localized disease, affecting the extremities and digits, is less severe. Rarely has the disease been found to affect the face, shoulder, genitalia and internal organs. The lesions rapidly progress and translate into necrotic non-healing ulcers and wet gangrene, which tend to get super infected. Mortality rates double when lesions ulcerate, reaching as high as 80% [4]. The leading cause of death at this point becomes sepsis and skin necrosis [8-12].

### Diagnosis

Many medical conditions can masquerade CUA, especially when the disease is distally localized involving the extremities. Differential diagnoses of CUA are highlighted in Table 2. Diagnosis is usually based on a triad of findings: characteristic cutaneous features in a uremic patient with abnormal biochemistry.

**Table 2:** Differential diagnosis of Calciphylaxis.

**Disease**	**Distribution**	**Chief characteristics**
**Peripheral Vascular Disease**	Lower extremities	Absent pulses, abnormal ankle brachial pressure index

**Venous ulcer**	Above the malleoli	Reddish brown discoloration with brawny edema suggesting venous stasis

**Leucocytoclastic vasculitis**	Symmetric, lower extremities	Palpable purpura evolve into hemorrhagic papules with milder symptoms; Positive serology/cryoglobulins; Characteristic skin biopsy

**Pyoderma gangrenosum**	Lower extremities	Associated with IBD and malignancy; Starts as a pustule, enlarges into violaceous erythematous plaque which ulcerates with heaped-up borders

**Disseminated Intravascular Coagulation**	Generalized	Widespread rapidly developing purpura. Associated with shock or disseminated infection and multi organ failure; A consumption coagulopathy

**Warfarin-induced skin necrosis**	Extremities, breast, trunk	History of warfarin use; May have associated protein C and S deficiency; Starts as erythematous macules which become edematous and necrotic; Skin biopsy shows fibrin thrombi within the blood vessels with interstitial hemorrhage

**Atheroembolic phenomena**	Diffuse	Starts as purpura after vascular procedure such as angiography

**Nephrogenic systemic fibrosis**	Symmetrical, extremities	Commonly associated with end stage renal disease on dialysis; May be associated with Gadolinium contrast agent; Presents as papules or plaques – areas of thick hardened skin with hyperpigmentation; Biopsy shows increased collagen bundles and fibroblast like cells

**Hypercoagulable states e.g. Protein C & S deficiency**	Trunk and extremities	History of thromboembolic events; Skin necrosis develops after initiating warfarin; Ecchymotic skin lesions coalesce and ulcerate leading to necrosis

Laboratory workup frequently shows hypercalcemia, hyperphosphatemia, and increased parathyroid hormone levels. To complete the workup, it is recommended to obtain coagulation studies, including prothrombin time (PT), partial thromboplastin time (PTT) and protein C and S levels to rule out hypercoagulable state. Autoimmune workup may be needed also to rule out vasculites. Imaging studies are not always helpful in diagnosis of CUA. Plain radiographs and computed tomography may show linear calcification of vessels. This finding is nonspecific and is commonly encountered in diabetes and ESRD. Xeroradiography, a high-definition x-ray photography that tends to improve structure visibility, is the best imaging to study soft tissue calcification [13]. Bone scan is positive in majority of patients with CUA and can be used to assess response to treatment. Trans-cutaneous oxygen saturation is low and may aid in the diagnosis of CUA [14]. Biopsy of skin lesion shows calcium deposition in tunica media of small and medium sized arteries and arterioles with intimal proliferation, lobular fat necrosis with infiltration of neutrophils, lymphocytes and macrophages. However biopsy is needed only in equivocal cases because of the risk of biopsy itself leading to development of non-healing ulcers.

### Treatment

Early diagnosis of CUA is necessary to reduce morbidity and mortality. Management is chiefly supportive and multidisciplinary. Triggering agents such as warfarin, corticosteroids, calcium, vitamin D, parenteral iron therapy should be discontinued. If levels of calcium and phosphate and, subsequently Ca × PO_4 _product are elevated, they should be normalized with dietary restriction of phosphorus through low protein diet and usage of calcium free phosphate binders such as sevelamer hydrochloride [15-17]. Increasing the frequency of dialysis, using a low calcium dialysate, is also recommended. Calcimimetics such as cinacalcet can help reduce parathyroid hormone levels, and hence calcium and phosphorus, in secondary hyperparathyroidism by increasing the sensitivity of calcium sensing receptors in parathyroid gland [18]. Bisphosphonates, sodium thiosulphate and hyperbaric oxygen reduce pain and hasten healing [19-21]. Parathyroidectomy is indicated in secondary hyperparathyroidism with CUA [22]. Pain relief with large doses of analgesics and lumbar sympathetic blockade may be necessary. Serial debridement of infected necrotic tissue and broad-spectrum antibiotics aid in wound healing and treating sepsis.

## Conclusion

Calciphylaxis is a rare but fatal disease. Hence great emphasis needs to be placed on prevention. Patients need to be educated regarding the importance of compliance with medications, dialysis, and proper diet and weight reduction. Avoiding the triggering factors and maintaining normal levels of calcium, phosphate and Ca × PO_4 _product seems to be the key, although CUA can occasionally occur in the absence of renal disease and a normal Ca × PO_4 _product [23-26].

## Competing interests

The authors declare that they have no competing interests.

## Authors' contributions

Both authors are involved in writing this case report.

## Consent section

Written informed consent was obtained from the patient for publication of this case report and accompanying images. A copy of the written consent is available for review by the Editor-in-Chief of this journal.
